# Missing Links in Predicting Berry Sunburn in Future Vineyards

**DOI:** 10.3389/fpls.2021.715906

**Published:** 2021-10-12

**Authors:** Christopher Bahr, Dominik Schmidt, Katrin Kahlen

**Affiliations:** Department of Modeling and Systems Analysis, Hochschule Geisenheim University, Geisenheim, Germany

**Keywords:** climate change, grapevine, heat, canopy architecture, light, functional-structural plant model

## Abstract

Sunburn in grapevine berries is known as a recurring disorder causing severe yield losses and a decline in berry quality. The transition from healthy to sunburnt along a temporal trajectory is not fully understood. It is driven by light-boosted local heat impact and modulated by, e.g., past environments of the berry and its developmental state. Events of berry sunburn are often associated with heatwaves, indicating a link to climate change. In addition, the sensitivity of grapevine architecture to changing environmental condition indicates an urgent need to investigate and adapt mitigation strategies of berry sunburn in future vineyards. In this perspective, we want to identify missing links in predicting berry sunburn in vineyards and propose a modeling framework that may help us to investigate berry sunburn in future vineyards. For this, we propose to address open issues in both developing a model of berry sunburn and considering dynamic canopy growth, and canopy interaction with the environment and plant management such as shoot positioning or leaf removal. Because local environmental conditions drive sunburn, we aim at showing that identifying sunburn-reducing strategies in a vineyard under future environmental conditions can be supported by a modeling approach that integrates effects of management practices over time and takes grapevine architecture explicitly into account. We argue that functional-structural plant models may address such complex tasks. Once open issues are solved, they might be a promising tool to advance our knowledge on reducing risks of berry sunburn *in silico*.

## 1. Introduction

Berry sunburn in grapevines is a recurring disorder that can reduce berry quality and cause severe yield loss (Keller, 2015). Recently, Gambetta et al. (2021) reviewed current knowledge on berry sunburn in grapevine. They conclude that processes resulting in sunburn are highly complex and not fully understood, but key drivers of sunburn are local light conditions and heat impact on the berry surface and a cultivar-specific susceptibility of the berry to sunburn. The latter may depend on various characteristics of the berry such as its developmental stage and its adaptation to the environment.

An increased emergence of sunburn has been observed in recent years in some vine regions in France and Germany (Gambetta et al., 2021). Given that berry sunburn is driven by extreme heat, more frequent and intense heatwaves, which can be expected in future (Masson-Delmotte et al., 2021), could indicate a link of climate change and sunburn. Thus, a more frequent occurrence of sunburn could be expected in the future (Silvestre et al., 2019; Santos et al., 2020; Gambetta et al., 2021), but only if viticulturist could not fully adapt canopy management and associated practices. Yet, we think that climate change might have even more significant effects on sunburn patterns in a future vineyard: Climate change might further advance phenological phases (Duchêne et al., 2010; Bernardo et al., 2018) and, e.g., shift the ripening phase into periods with higher temperatures, for example, in European and Australian wine regions (Jones et al., 2005; Webb et al., 2007, 2012). In the ripening phase, berries are particularly susceptible to sunburn (Bondada and Keller, 2012); thus, climate change might aggravate sunburn risks of newly sun-exposed berries in this phase. Being less susceptible to sunburn in earlier phases (Hulands et al., 2014) does not mean that there is no potential sunburn risk. Extreme temperatures in heatwaves might counterbalance the protective trait. Thus, assuming that climate change intensifies extreme events (Perkins-Kirkpatrick and Lewis, 2020), this might add sunburn-risk periods even to the earlier growth season. Then again, elevated CO_2_ (eCO_2_), one driver of climate change, may change bunch architecture (i.e., longer bunches), which might affect sun exposure, and increase growth of secondary lateral shoots, but periods of high temperatures may weaken this effect (Wohlfahrt et al., 2018). Obviously, both statements neglect effects of adapted management practices (Stoll et al., 2010; Zheng et al., 2017; Gatti et al., 2018; Valentini et al., 2018, 2021; Bei et al., 2019; Lavado et al., 2019; Hunter et al., 2020; Gutiérrez-Gamboa et al., 2021; Martinez De Toda, 2021; Naulleau et al., 2021; Schäfer et al., 2021) and other limiting factors like reduced soil water availability (Lopes et al., 2018). Thus, eCO_2_ might reduce sunburn risks in the later season because of shading berries by increased lateral leaf area, but high temperatures might attenuate the positive effect. On the other hand, leaf removal is a common management practice (Palliotti et al., 2013; Pastore et al., 2013; Torres et al., 2021), for example, to influence grape composition or to reduce disease pressure (Zenoni et al., 2017; Tóth, 2020; O'Brien et al., 2021; VanderWeide et al., 2021). While timing, extent, and need for leaf removal depend on local environmental factors, opening up the canopy at some point is usually recommended for promoting wine quality (Frioni et al., 2017; Hickey and Wolf, 2019; Satisha and Somkuwar, 2019; Würz et al., 2020; O'Brien et al., 2021). Even though, early leaf removal in the bunch zone can allow berries to better adapt to sunlight and, thereby, reduce their susceptibility to sunburn (Gambetta et al., 2021), leaf removal events just before or during a heatwave might dramatically increase sunburn occurrence due to newly sun-exposed berries being insufficiently adapted to the risky environment (Hayman et al., 2012; Palliotti et al., 2014). Again, if we expect more heatwaves due to climate change, this would shorten and reduce the time windows of leaf removal for protection against sunburn. In addition, strategic decisions such as row orientation, cultivar choice, and trellis system might interplay with the above-mentioned scenarios (Palliotti, 2011; Hunter et al., 2016, 2017; Zheng et al., 2017; Bernardo et al., 2018; Leeuwen et al., 2019; Chopard et al., 2021; Kurtural and Fidelibus, 2021; Sargolzaei et al., 2021). For example, in north-south oriented rows sunburn occurs often just on the west side of the rows (Spayd et al., 2002; Gambetta et al., 2021) due to an unbalanced temperature distribution with heat peaks in the afternoon (Lopes et al., 2018; Strack et al., 2021). Therefore, in order to reduce sunburn risks in such vineyards, leaf removal is sometimes limited to the morning side (east) of the canopy. However, climate change might increase temperature to a point where the heat impact might cause sunburn on non-shaded berries on the morning side. Thus, climate change might increase the sunburn risk of hitherto low-risk berries and add new locations of possible sunburn occurrence to the grapevines.

**Figure 1:**
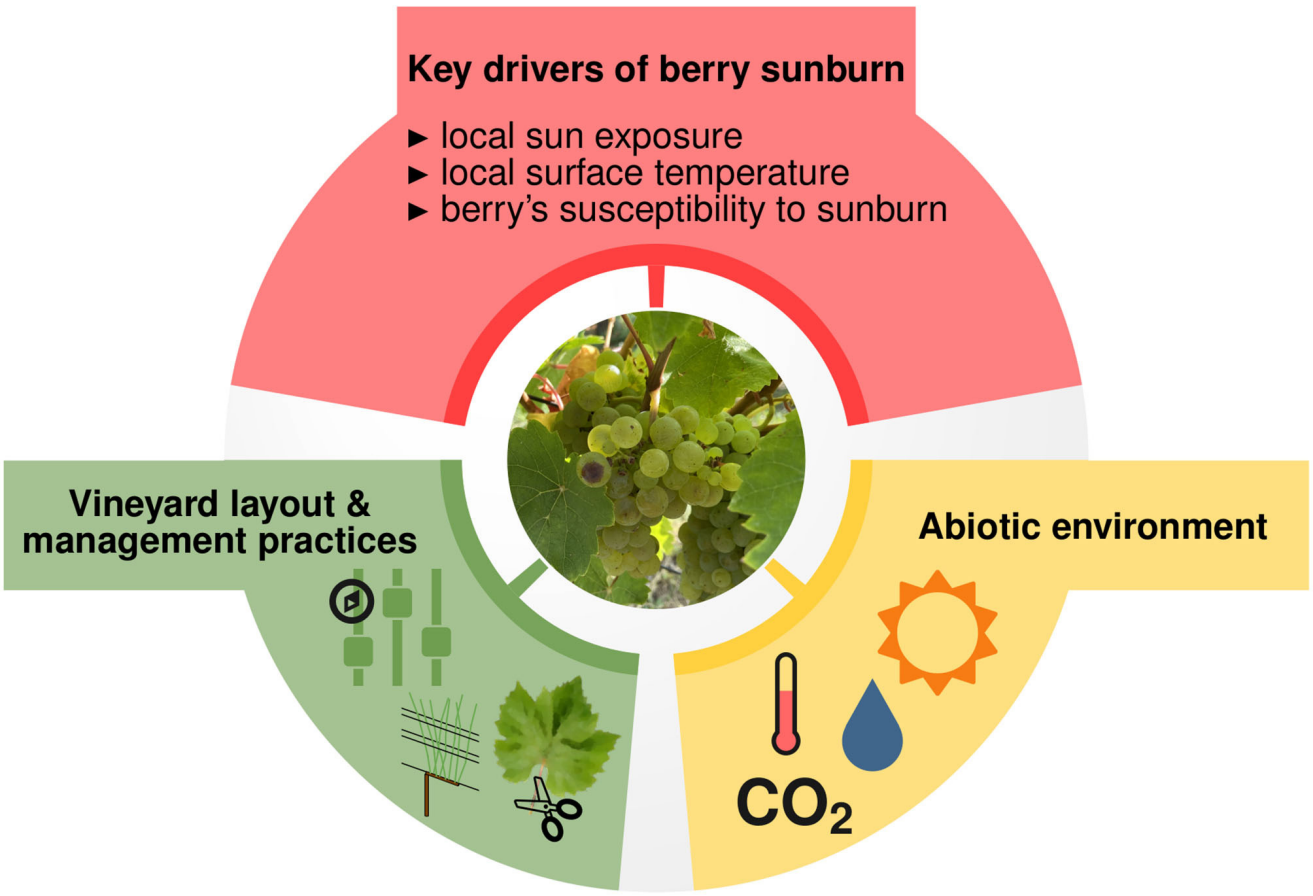
Sunburn in grapevine berries is a heat-induced disorder that requires direct exposure of the berries to the sun. Gambetta et al. (2021) suggested to predict the occurrence of berry sunburn from local sun exposure, local surface temperature, and the susceptibility of berry to sunburn. These traits are affected by environment and plant management. The abiotic environment can influence both heat impact on berries and vine architecture, thus local sun exposure. Plant architecture is also determined by the vineyard layout and management practices, such as shoot positioning and leaf removal, which in turn influences light penetration into the canopy and thereby affects sunburn risks.

In summary, the interplay of the discussed future climatic conditions, seasonal management practices, and strategic decisions on vineyard planning might severely affect future seasonal sunburn occurrence pattern. This underlines the importance of taking climate change explicitly into account when addressing sunburn in future vineyards. Hence, an advanced modeling tool for systematically analyzing future scenarios may then be needed to support and accelerate the development of adapted mitigation strategies. Recently, Gambetta et al. (2021) suggested predicting sunburn events based on the following modeling approach: “If the susceptibility of a given cultivar and developmental stage and the duration of adaptation were known, this information could be combined with accurate berry fruit surface temperature (FST) to predict sunburn events. In addition, modeling approaches on canopy level could provide a better insight for mitigation strategies of sunburn protection considering plant architecture and training systems in vineyards.” Figure 1 illustrates this idea.

In this perspective, we want to advance this idea and propose a modeling framework that may help us to investigate the extent of berry sunburn in future vineyards, while particularly considering climate change and pointing out missing links to be resolved for such a berry sunburn prediction. Other factors effecting canopy development and hence light penetration, such as nutrition and water status (Keller, 2005; Lopes et al., 2018; Briglia et al., 2020), are assumed to be unaltered and linked to a selected reference condition, although this limits the initial scope of the modeling framework.

## 2. Modeling Berry Sunburn of Grapevines

Following Gambetta et al. (2021), a model of berry sunburn of grapevine may assume that sunburn occurrence can be predicted from the following key characteristics of the berry: susceptibility of the given cultivar to sunburn, developmental stage, and duration of adaptation and berry surface temperature.

The output of the sunburn model for a berry is its sunburn state, either healthy or sunburnt. At the onset of berry and bunch growth, all berries can be assumed healthy. This could be reflected in the model by an initial sunburn value of SB = FALSE for all berries. During development, a berry either keeps this value or, if subjected to a sunburn event, its trait is set to SB = TRUE. For the decision of an irreversible state transition from healthy to sunburnt, the model could compare the surface temperature of the berry, FST, with a cultivar-specific threshold surface temperature *T*_*c*_. If the threshold surface temperature is exceeded, sunburn occurs. This can be expressed by


(1) $$\begin{array}{r} \text{ifFST}>T_{c}\;\text{then the sunburn state of berry:SB}=\text{TRUE}. \end{array}$$

The current susceptibility of a berry to sunburn could be expressed by a scaling factor *f*_*s*_ of the threshold temperature. This changes (Equation 1) as follows


(2) $$\begin{array}{r} \text{ifFST}>f_{s}\times T_{c}\;\text{then the sunburn state of berry:SB}=\text{TRUE}. \end{array}$$

Based on this approach, a decreasing *f*_*s*_ would cause sunburn at lower FST. If the factor *f*_*s*_ reflects both, the developmental stage of berry and duration of adaptation, the model could echo the changing susceptibility of a berry to sunburn. Such a modeling approach seems quite appealing at the first glance, because of its simplicity and close link to observations in the field. However, we need to overcome missing links before it would be applicable. These missing links are directly related to the model components in Equation (2) but also to the fact that berry sunburn is a disorder that requires direct sun exposure of the berries and, therefore, depends on the canopy architecture of the grapevines. The reason for this is that shaded berries typically do not show sunburn symptoms at all, since the required heat impact for sunburn is not supplied by ambient temperature alone (Gambetta et al., 2021).

**Cultivar-specific threshold temperature**, **T_*c*_**: Since the model should consider varying susceptibility of a berry to sunburn by accounting for the developmental stage of the berry and duration of adaptation, the cultivar-specific threshold temperature, *T*_*c*_, has to represent a reference condition. This reference condition could be a combination of developmental stage “véraison” (beginning of berry ripening) and lowest susceptibility of a berry to sunburn. In addition, to be useful as reference value in the model, *T*_*c*_ has to be a constant value. Yet, it is still an open task to show that *T*_*c*_ is such a robust trait to asses sunburn.

**Susceptibility factor**, _**f*S***_: To allow the comparison of temperatures in Equation (2), the susceptibility factor of a berry to sunburn has to be dimensionless and equal 1 for the reference condition of *T*_*c*_. In order to echo observations, *f*_*s*_ should depend on both, developmental stage (DS) and berry skin adaptation to sun exposure (SE). The following equation mimics a simplified modeling approach of this relation:


(3) $$\begin{array}{r} f_{s}=\text{function}\left(f_{\text{DS}},f_{\text{SE}}\right) \end{array}$$

where *f*_DS_ represents the dependency of sunburn susceptibility on the developmental stage of a berry, and *f*_SE_ includes variation in susceptibility with respect to the sustaining sun exposure of berry. Thus, *f*_DS_ should cause *f*_*s*_ to decrease with time from minimum susceptibility at the onset of berry growth to maximum susceptibility at harvest. In contrast, a minimum susceptibility should be reached at full sun adaptation reflected by a *f*_SE_ causing *f*_*s*_ to increase. However, response functions of the different aspects of berry susceptibility are unknown and it is unclear whether these aspects act independently from each other. For estimating parameters experimentally, first attempts have shown that grapes grown in different conditions can successfully be burnt applying artificial light and taking thermal images to determine a surface temperature (Müller et al., 2021).

**Berry surface temperature**, **FST**: For predicting berry sunburn in future vineyards, it would be necessary to predict berry surface temperature as well. Energy-balance models allow estimating FST of single berries grown in controlled conditions (Smart and Sinclair, 1976) and in the field (Cola et al., 2009; Ponce de León and Bailey, 2021). The model of Cola et al. (2009) is setup for red grapevine berries in a hedge-like row canopy from véraison to harvest and predicts FST from sensible heat flow, air temperature and a turbulent exchange coefficient using constant values of leaf area index and row height as input. This model estimates FST within static architectural conditions in the field sufficiently accurate for the model purpose (Cola et al., 2009). In contrast, the model of Ponce de León and Bailey (2021) successfully introduced a heat storage term for predicting rapid spatial and temporal fluctuations in berry temperature. The model is validated against experimental data and predicts average berry temperature with high accuracy (assessed by coefficients of determination above 94% and low errors). However, advancements are needed to make both models sensitive to changing canopy architecture caused by grapevine growth and interactions with the environment or plant manipulation events.

**Light-exposure of the berries**: Berry sunburn requires sun exposure, which, therefore, needs to be monitored as mandatory prerequisite of the above-described approach of modeling sunburn. However, the sheer number of berries in a vineyard does not permit tracking sun exposure of all berries in a vineyard simultaneously. It seems reasonable that predicting local light conditions on the berries could help to overcome these challenges. Certainly, the penetration of light into the grapevine canopy depends on many factors such as the trellis system (e.g., vertical shoot positioning), row spacing and orientation, leaf positioning within the canopy including the optical properties of canopy, but also plant management such as leaf removal (Zorer et al., 2017; Naulleau et al., 2021). A simple model of light attenuation within a canopy, such as Beer-Lambert equation, allows precise estimates in horizontally homogeneous canopies based on leaf area index and an experimentally derivable light extinction coefficient (Monsi and Saeki, 2005). However, such an approach does not result in accurate snapshots of local light conditions within a heterogeneous grapevine row canopy. As a consequence, for modeling sunburn in vineyards, model approaches are needed that echo canopy architecture and its interplay with the incoming light in high resolution.

## 3. Toward Predicting Berry Sunburn in Future Vineyards

A specific class of plant models, the so-called functional-structural plant models (FSPMs), can integrate structural components of a canopy in detail and can even catch the variability of canopy (e.g., Schmidt and Kahlen, 2019; Boudon et al., 2020). They explicitly combine plant architecture and plant functioning. FSPMs can be used to deal with research questions ranging from basic research to applied sciences (Louarn and Song, 2020). Understanding plant functioning across scales and integrating multidisciplinary knowledge remain an ambitious task in FSPMs (Louarn and Song, 2020), but they have particularly proven useful for addressing complex interactions of plants and their light environment (e.g., Kahlen and Stützel, 2011).

For grapevine, there already exist several FSPM approaches. Most of them consider canopy architecture in detail but focus on static snapshots of grapevine architecture captured by digitized real plants (e.g., Louarn et al., 2005). The pioneering model *Top-vine* simulates light-sensitive differences in the variability of canopy structure of cultivar × training system pairs for cvs. Grenache Noir and Syrah (Louarn et al., 2008). Follow-up models of *Top-vine* (Prietro et al., 2012; Prieto et al., 2019) and further grapevine-FSPMs (Zhu et al., 2018; Albasha et al., 2019) focus on linking complex physiological processes such as photosynthesis and transpiration to static architectural constraints: Prietro et al. (2012) adapted the architectural model of *Top-vine* to fit it to digitized data of a single grapevine cv. Syrah of each experimental site and used this model to examine the variability of gas exchange within the canopy, taking into account the nitrogen content of the leaves and the local adaptation to radiation in the grapevine. The latest development of this study highlights the role of N-distribution within the canopy on gas exchange of canopy architectures established by different training systems (Prieto et al., 2019). In the FSPM *GrapevineXL*, Zhu et al. (2018) linked local plant architecture to a bio-mechanical model of gas exchange and a water status model. They simulated berry quality based on carbon and water fluxes. In this study, the descriptive architecture mimicked the conditions of grapevine fruiting cuttings of cv. Cabernet Sauvignon in a greenhouse environment. The model *HydroShoot* is a FSPM that considers plant architecture for simulating transpiration and net photosynthesis rates at leaf and plant level of single grapevines (Albasha et al., 2019). To achieve this, *HydroShoot* does not take into account time-dependent changes in plant architecture.

So far, just a very few grapevine-FSPMs consider dynamic plant growth over the season (Garin et al., 2014; Schmidt et al., 2019). *Top-vine* data also served as the basis of the first dynamic grapevine-FSPM to analyze the development of powdery mildew (Garin et al., 2014). In contrast, *Virtual Riesling*, a FSPM for field-grown Riesling, was developed using repeatedly digitized vines grown in a unique vineyard facility established to catch climate change impact on grapevine (Schmidt et al., 2019). This model already allows assessing the role of changing temperatures in grapevine architecture and thereby considering management techniques such as vertical shoot positioning (Schmidt et al., 2019). Most recently, *Virtual Riesling* was coupled with a light model (Bahr et al., 2020) for analyzing the effects of leaf removal on light distribution within the canopy (Bahr et al., 2021) and it was initially calibrated for assessing the effects of elevated ambient CO_2_ concentrations on grapevine growth and development (Schmidt et al., 2020). However, the current version of *Virtual Riesling* does not include generative growth and an in-depth model evaluation is still missing. Recently, Ponce de León and Bailey (2021) developed a model for simulating single berry temperature in vineyards. Their canopies representing snapshots of four different trellis systems were built using a procedural plant model generator implemented in the software environment Helios (Bailey, 2019). Hence, this approach is mainly lacking of dynamic growth features for the canopy and the berries, to be applied in the proposed context of modeling berry sunburn (cf. Table 1). In summary, we conclude that all the above-mentioned grapevine-FSPMs require important advancements to allow for integrating a sunburn sub model and reliably predicting sunburn in future vineyards.

**Table 1:** Grapevine FSPMs, their original purpose, and necessary features listed to model berry sunburn.

**Grapevine FSPMs**			**Necessary features for berry sunburn modeling**				
Model^*^	**Purpose**	**Cultivar**	**Dynamic growth**	**In-season management**	**Berries**	**Berry growth**	**FST model**
*Top-vine^a^*	canopy structure × training system, gas-exchange × nitrogen content × radiation	Grenache Noir, Syrah	no	no	no	no	no
*Hydroshoot^b^*	gas-exchange × water deficit	Syrah	no	no	no	no	no
*GrapevineXL^c^*	berry growth × water flux × carbon flux	Cabernet Sauvignon	no	no	yes	yes	no
*Top-vine (OpenAlea)^d^*	powdery mildew development	n.a	yes	no	no	no	no
*Virtual Riesling^e^*	canopy structure × training system × plant management × light interception	Riesling	yes	yes	no	no	no
*Helios^f^*	berry temperature × training system	Cabernet Sauvignon	no	no	yes	no	yes

* *model specific information taken from cited publications*.

a *(Louarn et al., 2005, 2008; Prietro et al., 2012; Prieto et al., 2019)*.

b *(Albasha et al., 2019)*.

c *(Zhu et al., 2018)*.

d *(Garin et al., 2014)*.

e *(Schmidt et al., 2019; Bahr et al., 2021)*.

f *(Ponce de León and Bailey, 2021)*.

## 4. Toward *in silico* Experiments for Developing Mitigation Strategies of Sunburn Protection

An advanced grapevine-FSPM could be used to identify plant architectures and management strategies favorable for reducing sunburn risks under detrimental environments based on *in silico* experiments. Such *in silico* experiments are simulation studies that mimic real experiments. In other words, the advanced model would be used to simulate virtual vineyards including treatments and replications. From the *in silico* experiments, we could extract information on sunburn occurrence within virtual vineyards (location, time and probability) to identify correlations with characteristics from climatic measures (thermal course and radiation intensity), morphological measures (leaf area and bunch dimensions), phenological stages and applied management practices.

Before exploring future conditions *in silico*, an obligatory validation study comparing recent sunburn occurrence with simulated sunburn risks has to attest sufficient model accuracy. Simulations of vineyards responding to changes in environmental conditions should give us answers to the impact of climate change on sunburn. For this, a series of *in silico* experiments should be performed to estimate the effects of morphological responses to eCO_2_, increased temperature, and heatwaves on sunburn occurrence. Performing simulations with various options for management practices, such as timing, location, and intensity of leaf removal, under challenging environments would then allow us to identify optimized management practices for reducing sunburn. However, a model focusing exclusively on sunburn would not cover trade-offs between possible conflicting objectives of a viticulturist such as controlling sugar content or avoiding pests and other diseases (Santos et al., 2020). Thus, it would be of great advantage to apply newly identified strategies theoretically favorable for reducing sunburn risks in real vineyards to test their effect and also to reveal potential management conflicts (e.g., in *VineyardFACE* at Geisenheim University, Germany, e.g., Wohlfahrt et al., 2018). In addition, it could be necessary to advance the grapevine model to include further processes of interest, yet this is beyond the scope of this perspective. To summarize, we suggest to integrate a sunburn model into an advanced grapevine-FSPM, to conduct *in silico* experiments and use them to identify management strategies and plant architectures favorable for reducing sunburn risks in future vineyards, and to test them in the field.

Since almost all existing grapevine-FSPMs on vineyard level are based on data collected on a specific site, this reduces the transfer ability of any such advanced model to other sites or environmental conditions that were not considered for model development (Jones et al., 2017). Nevertheless, if the proposed approach proves to be valuable, further extensions (various varieties, scion-rootstock combinations, and cultivation methods) can follow.

## 5. Conclusion

Viticulture demands to control fruit quality and yield, while reducing pest, diseases, and disorders such as berry sunburn. Canopy management can reduce the risk of sunburn; however, climate change and particularly heatwaves might make it necessary to adapt strategies to the new environmental conditions. Sunburn events are results of the complex interplay of environment and grapevine architecture affecting both the local heat impact on the berry surface and the susceptibility of berry to sunburn. Accordingly, a modeling approach to predict sunburn in vineyards should consider plant architecture, environment, and their interaction over time. We suggest that functional-structural plant models can be appropriate tools to integrate these sunburn aspects. However, current grapevine-FSPMs require further advancements to allow for integrating a sunburn sub-model reliably predicting sunburn in future vineyards. In this perspective, we highlighted missing links that have to be addressed. These are related to a concept and the parametrization of a berry sunburn model, the model input of berry exposure to direct sunlight and the role of dynamics in plant growth, and plant canopy management and environment. Once open issues are solved, and the proposed modeling framework should help us to better understand how climate change may affect sunburn and, thus, could provide new ideas for mitigating effects of climate change.

## Data Availability Statement

The original contributions presented in the study are included in the article/supplementary material, further inquiries can be directed to the corresponding author.

## Author Contributions

KK and DS designed the overall concept. All authors wrote the manuscript and approved the final version and publication.

## Funding

We acknowledge support by the Deutsche Forschungsgemeinschaft (DFG, German Research Foundation), project numbers 449374897 and 432888308, and the Open Access Publishing Fund of Geisenheim University.

## Conflict of Interest

The authors declare that the research was conducted in the absence of any commercial or financial relationships that could be construed as a potential conflict of interest.

## Publisher's Note

All claims expressed in this article are solely those of the authors and do not necessarily represent those of their affiliated organizations, or those of the publisher, the editors and the reviewers. Any product that may be evaluated in this article, or claim that may be made by its manufacturer, is not guaranteed or endorsed by the publisher.
